# Gut microbiota, circulating inflammatory proteins and sepsis: a bi-directional Mendelian randomization study

**DOI:** 10.3389/fcimb.2024.1398756

**Published:** 2024-08-08

**Authors:** Zuming Li, Liangcai Lin, Yunqi Kong, Jieni Feng, Xiaolei Ren, Yushi Wang, Xueru Chen, Siyi Wu, Rongyuan Yang, Jiqiang Li, Yuntao Liu, Yue Lu, Jiankun Chen

**Affiliations:** ^1^ The Second Clinical Medical College, Guangzhou University of Chinese Medicine, Guangzhou, China; ^2^ The Third Clinical Medical College, Guangzhou Medical University, Guangzhou, China; ^3^ Guangdong Provincial People's Hospital, Guangzhou, China; ^4^ State Key Laboratory of Traditional Chinese Medicine Syndrome, The Second Affiliated Hospital of Guangzhou University of Chinese Medicine, Guangzhou, Guangdong, China; ^5^ The Second Affiliated Hospital (Guangdong Provincial Hospital of Chinese Medicine), Guangzhou University of Chinese Medicine, Guangzhou, Guangdong, China; ^6^ Guangdong Provincial Key Laboratory of Research on Emergency in TCM, Guangzhou, China; ^7^ State Key Laboratory of Dampness Syndrome of Chinese Medicine, The Second Affiliated Hospital of Guangzhou University of Chinese Medicine, Guangzhou, China; ^8^ Guangdong Provincial Key Laboratory of Clinical Research on Traditional Chinese Medicine Syndrome, Guangzhou, China

**Keywords:** circulating inflammatory proteins, genome-wide association study, gut microbiota, Mendelian randomization, sepsis

## Abstract

**Background:**

Gut microbiota is closely related to the occurrence and development of sepsis. However, the causal effects between the gut microbiota and sepsis, and whether circulating inflammatory proteins act as mediators, remain unclear.

**Methods:**

Gut microbiota, circulating inflammatory proteins, and four sepsis-related outcomes were identified from large-scale genome wide association studies (GWAS) summary data. Inverse Variance Weighted (IVW) was the primary statistical method. Additionally, we investigated whether circulating inflammatory proteins play a mediating role in the pathway from gut microbiota to the four sepsis-related outcomes.

**Results:**

There were 14 positive and 15 negative causal effects between genetic liability in the gut microbiota and four sepsis-related outcomes. Additionally, eight positive and four negative causal effects were observed between circulating inflammatory proteins and the four sepsis-related outcomes. Circulating inflammatory proteins do not act as mediators.

**Conclusions:**

Gut microbiota and circulating inflammatory proteins were causally associated with the four sepsis-related outcomes. However, circulating inflammatory proteins did not appear to mediate the pathway from gut microbiota to the four sepsis-related outcomes.

## 1 Introduction

Sepsis is an unusual systemic response to a common infection, representing a mode of immune system response to injury (Faix, 2013). It can cause symptoms in multiple organ systems, such as the heart, lungs, kidneys, and digestive system (Lelubre and Vincent, 2018). According to epidemiological studies, the prevalence of sepsis and the 28-day mortality rate in hospitals range from 25% to 30% (Cohen et al., 2015a). The hyper-inflammatory response is followed by a period of immunosuppression, during which patients develop multiple organ dysfunction and are prone to nosocomial infections (Faix, 2013). Despite its alarming prevalence and severe consequences, treatment options remain limited and have for many years revolve around antibiotics and supportive therapies (Gotts and Matthay, 2016).

Gut microecology, as a unit of interaction between the gut microbiota and the host, is closely related to human health, with gut microbiota being the most important (Alkasir et al., 2017). Intestinal flora refers to the various microbial communities living in the intestinal cavity that adhere to the intestinal mucosa, including Archaea and Eukaryotes, which are predominantly bacterial. They have evolved together with the host in symbiotic relationships and play important roles in substance metabolism, nutrition, and immune protection (O’Hara and Shanahan, 2006; Dang and Marsland, 2019). Previous studies have shown that gut microbiota is linked to sepsis (Zhang et al., 2023). Several well-established risk factors for sepsis such as aging, immunization, and antibiotic resistance are associated with significant changes in the composition and function of the gut microbiome (Takiishi et al., 2017; Ling et al., 2022; Santacroce et al., 2023). However, the extent to which gut microbes are associated with sepsis remains unclear. A better understanding of the causal effects of the gut microbiota and the potential mediators between them will provide evidence for further mechanistic and clinical studies on the management and treatment of sepsis. Additionally, gut microbiota plays an important role in regulating the circulating inflammatory proteins (Xue et al., 2023).

To the best of our knowledge, inflammation plays an important role in promoting sepsis. Previous studies have revealed that sepsis is a common complication of combat injuries and trauma and is defined as a life-threatening organ dysfunction caused by a dysregulated host response to infection (Liu et al., 2022). During the body’s response to infection, an inflammatory response is essential. We hypothesized that circulating inflammatory proteins might mediate the pathway from the gut microbiota to sepsis.

Although randomized controlled trials can help establish a causal relationship between gut microbiota or circulating inflammatory proteins and sepsis, they are difficult to perform in humans due to objective limitations, such as screening the gut microbiota and elevating circulating inflammatory protein levels (Cheng et al., 2023; Combrink et al., 2023). Therefore, most current research conclusions are based on observations of composition and changes in the intestinal flora in the feces of patients with sepsis. Some observational cohort studies have shown that gut microbiota may be associated with sepsis (Deng et al., 2023; Park et al., 2024). Preclinical studies have demonstrated that the gut microbiota plays a key role in the immune response to systemic inflammation, and disruption of this symbiotic relationship increases the susceptibility to sepsis (Haak et al., 2018).

Although probiotic supplementation has positive effects (Vulevic et al., 2015; Panigrahi et al., 2017; Shimizu et al., 2018), its effectiveness and safety remain controversial (Zhang et al., 2023). Therefore, further research is required to determine the specificity and safety of these probiotic supplements. Mendelian randomization (MR) is an epidemiological method that uses genetic variation as an instrumental variable for exposure to estimate the causal effect of exposure on outcomes and strengthens causal inference. It overcomes the limitations of traditional observational study designs by using Mendelian laws, exploiting the random assignment to genotypes at conception, making genotypes independent of potential confounders, and avoiding reverse causality (Smith et al., 2007). Two-sample MR analysis is an extension of the MR approach that allows summary statistics from genome-wide association studies (GWAS) to be used in MR studies without the direct analysis of individual-level data (Davey Smith and Hemani, 2014). In recent years, two-sample MR studies have gradually been recognized by researchers, allowing data between genetic instrument variables and phenotypes, phenotypes, and diseases to come from two different independent populations, thereby improving the efficiency and statistical power of the study (Burgess et al., 2016; Gupta et al., 2017; Davies et al., 2018).

This study employed a comprehensive MR analysis to investigate the causal association between gut microbiota, circulating inflammatory proteins, and sepsis-related outcomes. Additionally, we examined the potential mediating role of circulating inflammatory proteins in the pathway from gut microbiota to sepsis. Furthermore, reverse causality analysis was conducted to assess the impact of genetic susceptibility to sepsis on gut microbiota and circulating inflammatory proteins.

## 2 Methods

### 2.1 Study design

**Figure 1:**
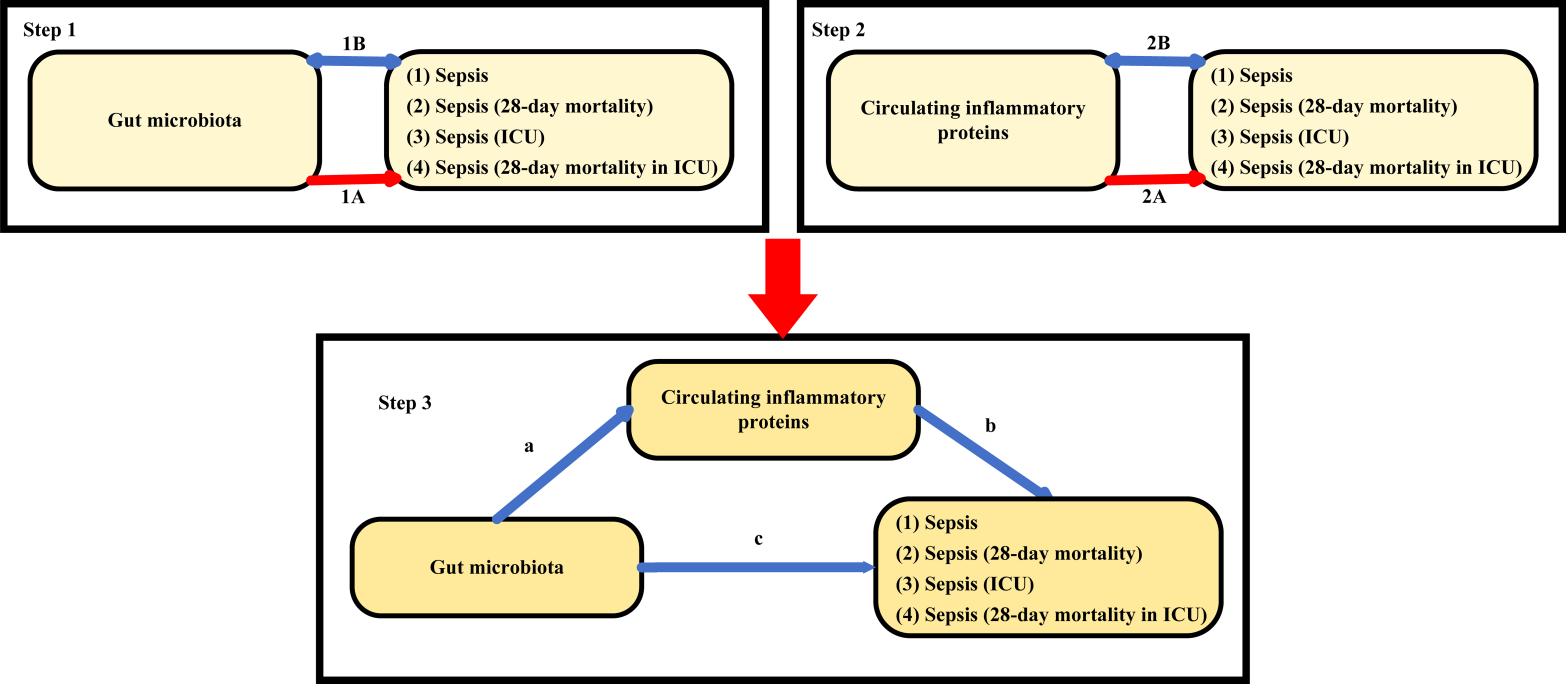
Study overview. Step 1A represents the causal effects of gut microbiota on four sepsis-related outcomes. Step 1B represents the bi−directional causal effects between gut microbiota and four sepsis-related outcomes. Step 2A represents the causal effects of circulating inflammatory proteins on four sepsis-related outcomes. Step 2B represents the bi−directional causal effects between circulating inflammatory proteins and four sepsis-related outcomes. Step 3 represents the mediating analysis of circulating inflammatory proteins in the pathway from the gut microbiota to four sepsis-related outcomes: path c was the total effect of gut microbiota on four sepsis-related outcomes; path b was the causal effect of circulating inflammatory proteins on four sepsis-related outcomes; path a was the causal effect of gut microbiota on circulating inflammatory proteins.

This study followed the STROBE-MR guidelines (Skrivankova et al., 2021) and key principles of the Strengthening Epidemiological Observational Research Reporting (STROBE) guidelines (von Elm et al., 2007). Detailed information is provided in **Supplementary Table S1**. As shown in **Figure 1**, this study consisted of three main components: analysis of the causal effects of 207 gut microbiota on four sepsis-related outcomes (step 1A), analysis of the causal effects of 91 circulating inflammatory proteins on four sepsis-related outcomes (step 2A), and mediation analysis of circulating inflammatory proteins in the pathway from gut microbiota to sepsis (step 3). In Mendelian randomization, single-nucleotide poly-morphisms (SNPs) are defined as instrumental variables (IVs). This approach is based on three core assumptions: (1) the IVs are closely associated with the exposure factors, (2) IVs are not associated with confounding factors, and (3) IVs do not affect the outcome directly and can only affect outcomes via exposure (Bowden and Holmes, 2019).

### 2.2 Data source

The Dutch Microbiome Project (DMP) provides summary data from the GWAS of 7,738 European individuals to obtain species-level data on the gut microbiota and maximize statistical power (Lopera-Maya et al., 2022). The participants in this database were relatively homogeneous, and it is currently the largest database of species-level gut microbiota data. Based on shotgun metagenomic sequencing of the stool samples, we identified 207 taxa (5 phyla, 10 classes, 13 orders, 26 families, 48 genera, and 105 species) in the gut microbiome. Genetic data for circulating inflammatory proteins were obtained from a previous GWAS (14,824 individuals). Circulating inflammatory proteins were measured using an Olink Target-96 Inflammation immunoassay plate, which measures 91 inflammation-related proteins (Zhao et al., 2023).

The main outcomes of this study were four sepsis-associated outcomes: the occurrence of sepsis, sepsis necessitating admission to critical care, mortality within 28 days in the intensive care unit (ICU) after experiencing sepsis, and sepsis 28-day mortality. In the Integrative Epidemiology Unit (IEU) OpenGWAS project database (https://gwas.mrcieu.ac.uk/) (Hemani et al., 2018), these outcomes were identified by the IDs ieu-b-4980, ieu-b-4982, ieu-b-4981 and ieu-b-5086, respectively. The Global Burden of Disease (GBD) study codes were used to define sepsis based on the International Classification of Diseases (ICD)-10 (Rudd et al., 2020).

This study was a secondary analysis of publicly available GWAS summary statistics. Ethical approval was granted to each original GWAS. Moreover, no individual-level data were used in this study. Therefore, no additional ethical review board approval was required.

### 2.3 Instrumental variables selection

First, we selected the SNPs with significant associations for gut microbiota (*P* < 1×10^–5^) (Shi et al., 2024). SNPs with significant associations with circulating inflammatory proteins were selected (*P* < 5×10^–8^) (Yan et al., 2024). We excluded the SNPs with linkage disequilibrium (LD) in the analysis. The LD of selected SNPs strongly related to the gut microbiota and circulating inflammatory proteins should meet the conditions of r^2^ < 0.001 and distance > 10,000 kb (Myers et al., 2020). After matching the outcomes, we removed the palindromic SNPs. SNPs significantly associated with the gut microbiota and circulating inflammatory proteins are listed in **Supplementary Tables S2** and **S3**.

To assess the strength of the relationship between the identified independent variables (IVs) and exposure, we computed the explained variance (R^2^) and F-statistic parameters. Steiger’s Test was used to detect and avoid reverse causality. The Steiger Test is a new methodology that enhances the robustness of the IV extraction (Hemani et al., 2017). SNPs with F-statistic parameters >10 were considered strong instruments (Burgess et al., 2017). R^2^ = 2 × EAF × (1-EAF) × β^2^, F = R^2^ × (N-2)/(1-R^2^) (Papadimitriou et al., 2020).

### 2.4 Primary analysis

To assess the causal impact of the gut microbiota and circulating inflammatory proteins on the four sepsis-related outcomes, we conducted a two-sample Mendelian randomization (MR) analysis for each outcome (step 1A and 2A in **Figure 1**). The Inverse Variance Weighted (IVW) approach was employed as the primary analytical method, while the Wald ratio test was used for features consisting solely of one independent variable (IV) (Burgess et al., 2013). The outcomes of the MR analysis were presented as odds ratios (ORs) accompanied by their respective 95% confidence intervals (CI). The findings were considered statistically significant when the P-value of the IVW method was below 0.05, and the IVW and MR-Egger estimates exhibited consistent directional effects (Ji et al., 2024).

### 2.5 Mediation analysis

Using a two-sample analysis (step 1A and 2A in **Figure 1**), the gut microbiota and circulating inflammatory proteins with significant causal effects on the four sepsis-related outcomes were included in the mediation analysis. As shown in **Figure 1**, we examined the possible causal impact of the gut microbiota on circulating inflammatory proteins (step 3, path a). If this is the case, we will perform multiple MR analyses to explore whether circulating inflammatory proteins are the mediating factors in the pathway from the gut microbiota to the four sepsis-related outcomes.

### 2.6 Bi−directional causality analysis

To evaluate bi-directional causation effects between gut microbiota, circulating inflammatory proteins, and four sepsis-related outcomes, we used four sepsis-related outcomes as “exposure” and gut microbiota or circulating inflammatory proteins associated with four sepsis-related outcomes as “outcome” (step 1B and step 2B in **Figure 1**). We selected SNPs that were significantly associated with four sepsis-related outcomes (*P* < 5×10^–8^) as IVs (Chen et al., 2024).

### 2.7 Sensitivity analysis

We performed Cochran’s Q test to evaluate the heterogeneity of each SNP (Cohen et al., 2015b) and generated scatter plots of SNP exposure and outcome associations to visualize the MR results. Leave-one-out analysis was performed to evaluate whether each SNP affected the results (Burgess and Thompson, 2017). In addition, we use MR-PRESSO and MR-Egger regressions to test for the potential horizontal pleiotropy effect. MR-PRESSO was used to detect significant outliers and correct for horizontal plural effects by removing outliers (Verbanck et al., 2018). All analyses were performed using R (v4.3.0) statistical software. MR analysis was performed using the “TwoSampleMR” package. The “MR_PRESSO” package was used for multiplicity tests (Ong and MacGregor, 2019).

## 3 Results

### 3.1 Causal effects of gut microbiota and circulating inflammatory proteins on four sepsis-related outcomes

#### 3.1.1 Sepsis

**Figure 2:**
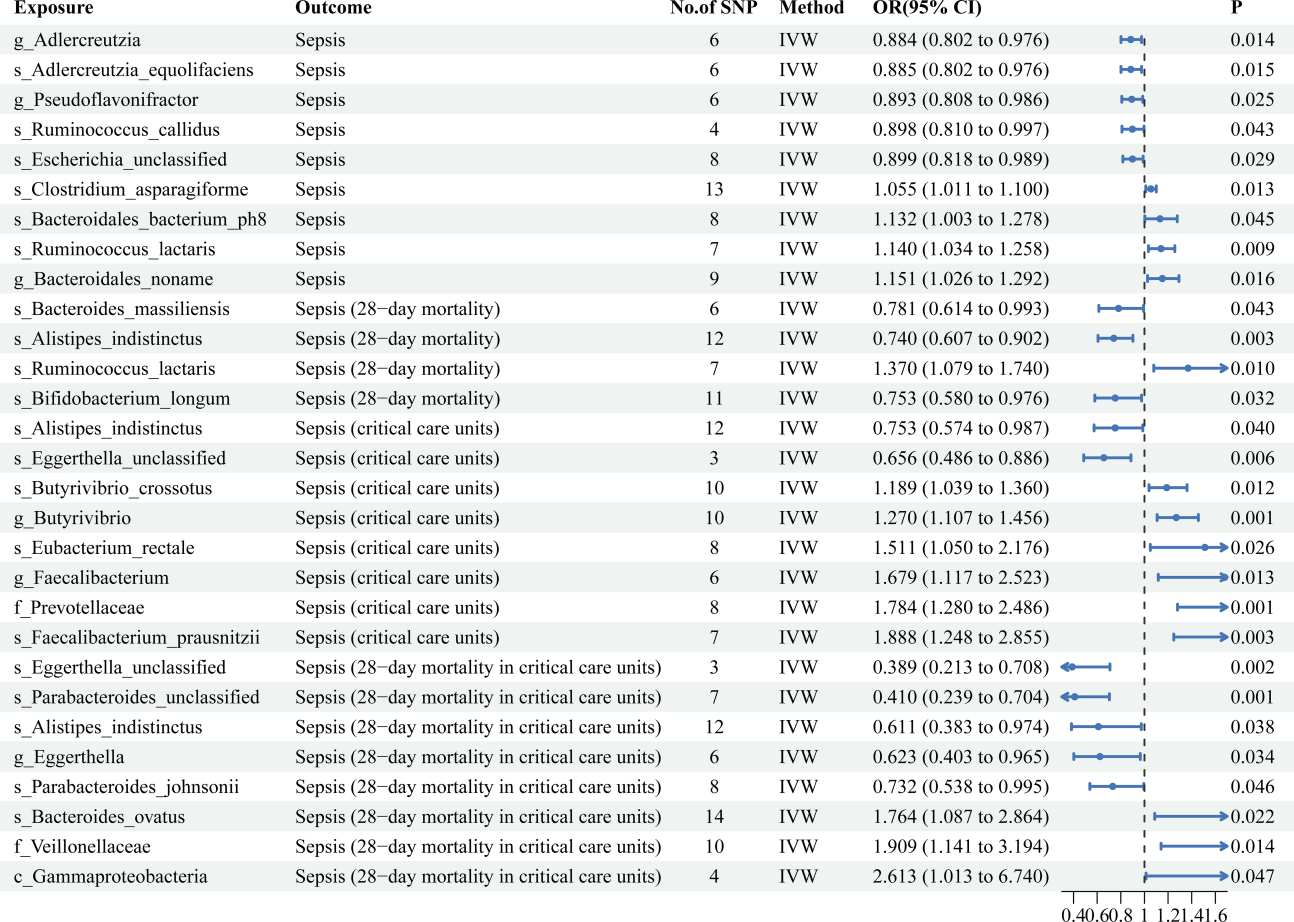
Mendelian randomization results of causal effects between gut microbiotas and four sepsis-related outcomes.

As shown in **Figure 2**, MR analysis suggested that the genetic prediction of four gut microbiota (species *Clostridium asparagiforme*, species *Bacteroidales bacterium ph8*, species *Ruminococcus lactaris*, and genus *Bacteroidale*) was associated with an increased risk of sepsis. Genetic prediction of five gut microbiota (genus *Adlercreutzia*, species *Adlercreutzia equolifaciens*, genus *Pseudoflavonifractor*, species *Ruminococcus callidus*, and species *Escherichia unclassified*) was associated with a decreased risk of sepsis (**Supplementary Table S4**).

**Figure 3:**
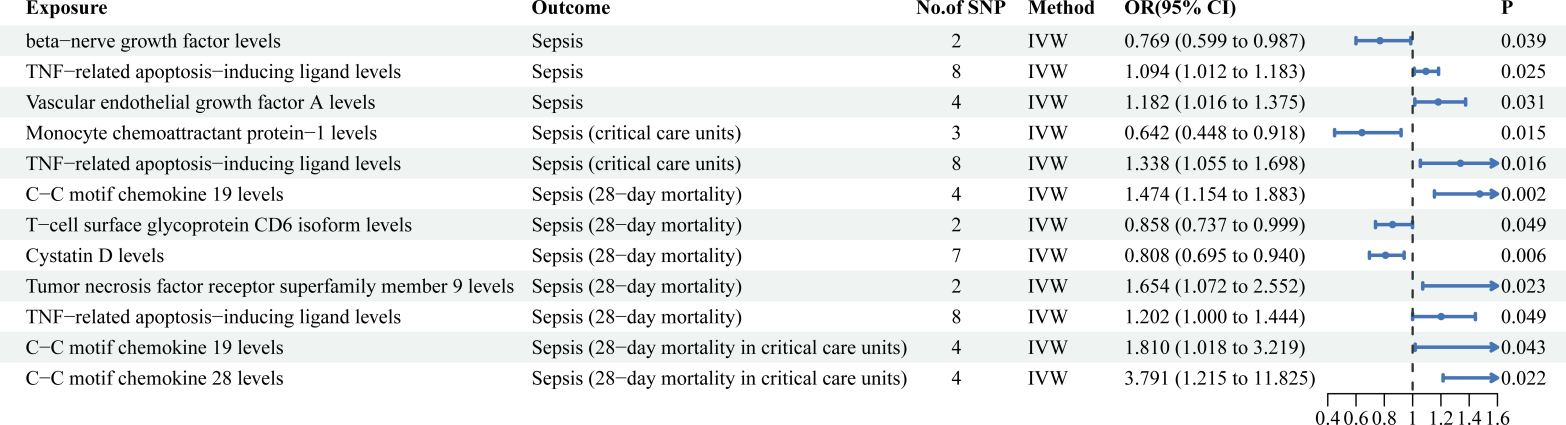
Mendelian randomization results of causal effects between circulating inflammatory proteins and four sepsis-related outcomes.

As shown in **Figure 3**, TNF-related apoptosis-inducing ligand levels (TRAIL) (OR = 1.094, 95%CI = 1.012 ~ 1.183, *P* = 0.025) and vascular endothelial growth factor A levels (VEGF-A) (OR = 1.182, 95%CI = 1.016 ~ 1.375, *P* = 0.031) were associated with an increased risk of sepsis. Beta-nerve growth factor levels (β-NGF) (OR = 0.769, 95%CI = 0.599 ~ 0.987, *P* = 0.039) significantly decreased the incidence of sepsis (**Supplementary Table S4**).

#### 3.1.2 Sepsis (28-day mortality)

As shown in **Figure 2**, the MR analysis suggested that the genetic prediction of one gut microbiota (species *Ruminococcus lactaris*) was associated with an increased risk of sepsis (28-day mortality). Genetic prediction of three gut microbiota (species *Bacteroides massiliensis*, species *Alistipes indistinctus*, and species *Bifidobacterium longum*) was associated with a decreased risk of sepsis (28-day mortality) (**Supplementary Table S4**).

As shown in **Figure 3**, C-C motif chemokine 19 levels (CCL19) (OR = 1.474, 95% CI = 1.154 ~ 1.883, *P* = 0.002), Tumor necrosis factor receptor superfamily member 9 levels (TNFRSF9) (OR = 1.654, 95%CI = 1.071 ~ 2.552, *P* = 0.023) and TRAIL (OR = 1.202, 95% CI = 1.000 ~ 1.444, *P* = 0.049) were associated with an increased risk of sepsis (28-day mortality). T-cell surface glycoprotein CD6 isoform levels (CD6) (OR = 0.858, 95% CI = 0.737 ~ 0.999, *P* = 0.049) and Cystatin D levels (OR = 0.808, 95% CI = 0.695 ~ 0.940, *P* = 0.006) were associated with a decreased risk of sepsis (28-day mortality) (**Supplementary Table S4**).

#### 3.1.3 Sepsis (critical care units)

As shown in **Figure 2**, MR analysis suggested that the genetic prediction of six gut microbiota (species *Butyrivibrio crossotus*, family *Prevotellaceae*, species *Eubacterium rectale*, species *Faecalibacterium prausnitzii*, genus *Butyrivibrio*, and genus *Faecalibacterium*) was associated with an increased risk of sepsis (critical care units). Genetic prediction of two gut microbiota (species *Alistipes indistinctus* and species *Eggerthella unclassified*) was associated with a decreased risk of sepsis (critical care units) (**Supplementary Table S4**).

As shown in **Figure 3**, TRAIL (OR = 1.338, 95% CI = 1. 055 ~ 1.698, *P* = 0.016) were associated with an increased risk of sepsis (critical care units). Monocyte chemoattractant protein-1 levels (MCP-1) (OR = 0.642, 95% CI = 0. 448 ~ 0.918, *P* = 0.015) were associated with a decreased risk of sepsis (critical care units) (**Supplementary Table S4**).

#### 3.1.4 Sepsis (28-day mortality in critical care units)

As shown in **Figure 2**, MR analysis suggested that the genetic prediction of the three gut microbiota (species *Bacteroides ovatus*, family *Veillonellaceae*, and class *Gammaproteobacteria*) was associated with an increased risk of sepsis (28-day mortality in critical care units). Moreover, all three factors significantly increased the risk of sepsis (28-day mortality in critical care units). Genetic prediction of five gut microbiota (species *Eggerthella unclassified*, species *Parabacteroides unclassified*, species *Alistipes indistinctus*, genus *Eggerthella*, and species *Parabacteroides johnsonii*) was associated with a decreased risk of sepsis (28-day mortality in critical care units). Moreover, all five significantly reduce the risk of sepsis development (28-day mortality in critical care units) (**Supplementary Table S4**).

As shown in **Figure 3**, CCL19 (OR = 1.810, 95% CI = 1.018 ~ 3.219, *P* = 0.043) and C-C motif chemokine 28 levels (CCL28) (OR = 3.791, 95% CI = 1.215 ~ 11.825, *P* = 0.022) significantly increased risk of sepsis (28-day mortality in critical care units) (**Supplementary Table S4**).

By comparing the results horizontally and vertically, interesting and surprising findings were obtained. In the gut microbiota, species *Ruminococcus lactaris* not only increased the risk of sepsis incidence but was also strongly associated with sepsis 28-day mortality. Although species *Alistipes inditinctus* had no significant effect on the incidence of sepsis, it had significant causal effects on sepsis (28-day mortality), sepsis (critical care units), and sepsis (28-day mortality in critical care units). Additionally, species *Eggerthella* had significant causal effects on the risk reduction of both sepsis (critical care units) and sepsis (28-day mortality in critical care units). Therefore, regulating species *Alistipes indistintus* and *Eggerthella* may be an important measure to prevent and treat critical sepsis. Among circulating inflammatory proteins, CCL19 not only increased the risk of sepsis (28-day mortality), but was also strongly associated with sepsis (28-day mortality in critical care units). TRAIL had significant effects on multiple sepsis-related outcomes.

### 3.2 Sensitivity analyses

Based on the MR-Egger regression intercept method, the outcomes were not influenced by genetic pleiotropy, and further analysis using MR-PRESSO demonstrated the absence of horizontal pleiotropy in the MR study (**Supplementary Tables S5, S6**). Cochran’s Q test revealed no significant heterogeneity (**Supplementary Table S7**). The findings from the “leave-one-out” analysis provide evidence supporting the reliability of MR analysis (**Supplementary Figures S1-S5**).

### 3.3 Bi−directional causal effects of four sepsis-related outcomes on gut microbiota and circulating inflammatory proteins

A P-value threshold of less than 5×10^-8^ was employed as the criterion to identify SNPs that exhibit significant associations with the three types of sepsis. However, no statistically significant SNPs were identified, indicating that the presence of a reverse causal effect was not substantial.

### 3.4 Mediation analysis

This study elucidated the causal relationships between gut microbiota, circulating inflammatory proteins, and four sepsis-related outcomes. Circulating inflammatory proteins appear to play a mediating role in the pathway from the gut microbiota to the four sepsis-related outcomes. One of the prerequisites for establishing a mediating effect is a significant association between the gut microbiota and circulating inflammatory proteins. Class *Gammaproteobacteria* (OR = 1.166, 95% CI = 1.010 ~ 1.345, *P* = 0.035) significantly increased beta-nerve growth factor levels. Species *Faecalibacterium prausnitzii* (OR = 0.861, 95% CI = 0.765 ~ 0.968, *P* = 0.012) significantly decreased C-C motif chemokine 19 levels (step 3a in **Figure 1**; **Supplementary Table S8**). However, the mediation analysis results showed that beta-nerve growth factor and C-C motif chemokine 19 could not mediate the influence of gut microbiota on sepsis-related outcomes, indicating that circulating inflammatory proteins did not act as mediators in the pathway from gut microbiota and four sepsis-related outcomes.

## 4 Discussion

In this study, we evaluated the bidirectional association between the gut microbiota and sepsis and investigated whether circulating inflammatory proteins act as mediators. We found 14 positive and 15 negative causal effects between genetic liability in the gut microbiota and four sepsis-related outcomes. Additionally, eight positive and four negative causal effects were observed between circulating inflammatory proteins and four sepsis-related outcomes. Circulating inflammatory proteins do not act as mediators. Notably, despite these findings, our reverse transcription analysis did not indicate any influence of sepsis on the gut microbiota or circulating inflammatory proteins.

There is a close relationship between the gut microbiota and sepsis. First, before the onset of sepsis, alterations in the gut microbiome increase the susceptibility to sepsis through multiple mechanisms, including (1) allowing the expansion of pathogenic gut bacteria, (2) providing a robust proinflammatory response to the immune system, and (3) reducing the production of beneficial microbial products, such as short-chain fatty acids (Adelman et al., 2020). Pathogenic bacteria present in the gut of healthy hosts may fail to proliferate and cause diseases in the presence of protective commensals (Chow et al., 2011). However, when protective bacterial taxa are lost, pathogens can proliferate and cause diseases (Kim et al., 2019; Woodworth et al., 2019). Previous studies have demonstrated the role of the gut microbiome in the initiation of the immune system in response to sepsis. After an episode of sepsis, changes in the gut microbiome can affect the inflammatory responses. Clinical studies on sepsis have shown a link between alterations in the gut microbiome, characterised by an increase in pathogenic bacteria and a robust immune response (Li et al., 2015; Cernada et al., 2016; Singer et al., 2018). In this study, we identified several gut microbiota that showed protective effects against sepsis, including species *Alistipes indistintus* and species *Eggerthella*. However, some taxa show potentially harmful effects, such as species *Eubacterium rectale* and genus *Bacteroidales*. Additionally, species *Ruminococcus lactaris* not only increased the risk of sepsis but was also strongly associated with sepsis mortality at 28 days.

*Alistipes* is a Gram-negative bacterium belonging to the phylum Bacteroides (Parker et al., 2020). Recent studies suggest that *Alistipes* may have protective effects against certain diseases, including colitis (Dziarski et al., 2016), liver fibrosis (Rau et al., 2018) and cardiovascular diseases (Kim et al., 2018; Zuo et al., 2019). The co-organisms of the intestinal microbiome produce short-chain fatty acids (SCFAs), which have the immune function of regulating the intestinal microenvironment. SCFAs play important roles in epithelial cell function. *Alistipes* is an acetate producer, and since previous studies have shown that short-chain fatty acids have an anti-inflammatory mechanism, it can be shown that *Alistipes* reduction promotes short-chain fatty acid reduction (Parker et al., 2020). *Eggerthella* is a bacterium that colonizes the human intestinal tract, female genital tract, oral cavity, and prostate (Lau et al., 2004). Recently, a growing body of evidence has collectively emphasized the association of *Eggerthella* with various diseases, such as asthma (Wang et al., 2018), multiple sclerosis (Cekanaviciute et al., 2017), systemic lupus erythematosus (Xiang et al., 2021), rheumatoid arthritis (Chen et al., 2016), and sepsis (Priputnevich et al., 2021). *Eggerthella* is likely to be implicated in sepsis; however, the exact role of *Eggerthella* in sepsis needs to be further explored. *Eubacterium* plays important roles in various processes, including the conversion of bile acids and cholesterol, metabolizing oxalate, producing anti-inflammatory molecules, reducing allergic airway inflammation, regulating insulin secretion, and controlling lipid metabolism (Mukherjee et al., 2020; Kamel et al., 2021). *Ruminococcus* plays an important role in the digestive process, especially in the decomposition and fermentation of plant fibers, such as cellulose and hemicellulose (Crost et al., 2023). *Ruminococcus* has been associated with various gastrointestinal, immune-related, and neurological disorders (Crost et al., 2023). Some studies have found that the number and activity of *Ruminococcus* in the guts of patients with these diseases may change, thereby affecting gut health and inflammation levels (Hall et al., 2017). *Bacteroides* are present in the human gut and have a symbiotic relationship with humans. They help break down food and produce the nutrients and energy required by the body. Specific taxa and their relative abundance in this phylum are associated with various diseases, including metabolic syndrome (Turnbaugh et al., 2009; Pedersen et al., 2016; Vieira-Silva et al., 2020), viral infections (Stefan et al., 2020). These conditions can cause or exacerbate sepsis. An experimental study indicated that in sepsis models, the dominant bacterial population in the lungs was *Bacteroides* (Dickson et al., 2016). And the relative abundance of *Bacteroides* in the lung microbiome was significantly correlated with serum TNF-α concentrations, a key mediator of the septic stress response that predicts sepsis patient mortality (Osuchowski et al., 2006). In some cases, *Bacteroides* may cause sepsis (Arnold et al., 2020). Perforations or ruptures of the intestine, dysbiosis of the intestinal microbiota, and impaired immune function allow intestinal bacteria such as *Bacteroides* to enter the abdominal cavity or bloodstream, causing infection and sepsis. More research is needed to fully understand the connection between gut microbiota and diseases, especially infection-related diseases such as sepsis.

This study aimed to determine whether the gut microbiota has a positive or negative impact on sepsis outcomes. However, the specific mechanism by which gut microbiota contribute to or worsen sepsis remains unclear. We hypothesized that inflammatory proteins in the bloodstream may play a role in the relationship between the gut microbiota and sepsis.

According to the MR analysis, we found that C-C motif chemokine 19 (CCL19), C-C motif chemokine 28 (CCL28), TNF-related apoptosis-inducing ligand (TRAIL), and vascular endothelial growth factor A (VEGF-A) levels were associated with an increased risk of sepsis-related outcomes. CCL19 and CCL28 are chemokines found in immune cells that play important immune-regulatory roles in the human body. Studies have shown a significant increase in plasma CCL19 levels in patients with sepsis, which may be related to sepsis severity (Tuerxun et al., 2023). However, research on the relationship between CCL19 and CCL28 and sepsis is currently limited. We look forward to a more comprehensive and in-depth research in the future. TRAIL, a biomarker of circulating cell death, is a potent inducer of apoptosis. Previous studies have shown that TRAIL has therapeutic effects in sepsis, peritonitis, and pulmonary inflammation (McGrath et al., 2011). Lower versus higher levels of TRAIL were associated with increased organ dysfunction and septic shock (Schenck et al., 2019). TRAIL is known to exert pluripotent effects in sepsis, leading to an improved survival rate in the early stages of abdominal sepsis and a decreased survival rate in the late phase of hypoinflammation (Boomer et al., 2014; Berg et al., 2023). It is worth noting that in recent years, animal experiments have found that TRAIL mediates immune suppression in sepsis, and that anti-TRAIL antibodies have a protective effect on sepsis mice (Gurung et al., 2011). VEGFA belongs to the ligand-tyrosine kinase receptor system, is widely expressed in the vascular system, and participates in the development of normal vascular barriers. VEGFA belongs to the ligand-tyrosine kinase receptor system, is widely expressed in the vascular system, and participates in the development of normal vascular barriers. During sepsis, the expression of VEGF-A increases, leading to damage to the vascular endothelial barrier, tissue inflammation, vascular leakage, insufficient blood volume, tissue oedema, and changes in microcirculation flow, ultimately leading to MODS (Hauschildt et al., 2020).

The development and management of sepsis are multifactorial, and this study specifically examined microecological differences to understand this condition. It is essential to acknowledge the complexity of sepsis and the need for a comprehensive evaluation of its various factors to gain a thorough understanding. This study is the first to reveal a potential causal relationship between the species levels of gut microbiota and sepsis-related outcomes, with profound implications for public health and the management of individual patients with sepsis. These findings may help diagnose sepsis more accurately. Based on the presence of multiple gut microbiota, patients with sepsis can be screened and assessed for the risk of taking preventive measures to reduce the incidence of sepsis (Peng et al., 2024). In addition, this study contributes to a deeper understanding of the pathogenesis and pathological processes of sepsis, providing a scientific basis for formulating targeted public health policies and prevention and control measures. Alleviates symptoms of sepsis or facilitates recovery by adjusting the gut microbiota balance. Interventions targeting the gut microbiota are needed for more effective treatment of sepsis (Cai et al., 2022).

This study represents a comprehensive initial MR analysis that examined the causal association between the gut microbiome, circulating inflammatory proteins, and sepsis-related outcomes. This study had several strengths. First, we highlighted certain species that showed more significant associations with sepsis than with other microbial classes. Although these associations were not statistically significant after adjusting for multiple testing, they nevertheless constitute important preliminary observations and may indicate underlying biological phenomena. In addition, this study used multiple sensitivity analyses to enhance the reliability of the findings. The consistency of the MR-Egger method with the IVW method demonstrates the robustness of the results. Despite the wide confidence intervals in some results, the overall patterns of the associations remained consistent. Furthermore, we employed the MR-PRESSO technique to identify and exclude potential outliers that may have biased our findings, thereby improving reliability. Finally, given that both the study population and the population surveyed were of European origin, the possibility of bias due to population stratification was reduced.

However, our study has several limitations. First, the limited European population data on gut microbiota and circulating inflammatory proteins may have biased our results. Second, the number of loci associated with circulating inflammatory proteins was relatively small compared to those associated with sepsis and gut microbiota. Third, our Mendelian randomization study did not have access to individual-level data, such as sex, age, and disease severity, which limited the depth of our analysis.

## 5 Conclusion

In summary, our bidirectional Mendelian randomization study clearly demonstrated 29 causal effects between genetic liability in the gut microbiota and sepsis-related outcomes, whereas the reverse causality hypothesis did not hold. Notably, our findings indicated that circulating inflammatory proteins do not act as mediators. To gain a more nuanced understanding of the observed association between the gut microbiota and sepsis, future studies should focus on potential mechanistic pathways while also attempting to adjust for potential confounders, such as diet, lifestyle, and medications, given that these factors may have a greater impact on sepsis. Our work is an important step forward in explaining the relationship between gut microbiota and sepsis, but more microbiological and clinical studies are needed to validate and expand our findings. We hope that this research will contribute to the diagnosis, treatment, and development of drugs for sepsis.

## Data availability statement

The original contributions presented in the study are included in the article/**Supplementary Material**. Further inquiries can be directed to the corresponding authors. The summary data of genome-wide association studies (GWAS) used in this study can be obtained from the Integrative Epidemiology Unit (IEU) OpenGWAS project database (https://gwas.mrcieu.ac.uk/) and the GWAS catalog (https://www.ebi.ac.uk/gwas/).

## Author contributions

ZL: Software, Writing – original draft, Writing – review & editing, Formal analysis, Funding acquisition, Supervision. LL: Data curation, Formal analysis, Investigation, Writing – original draft. YK: Validation, Formal analysis, Methodology, Writing – review & editing. JF: Data curation, Investigation, Writing – original draft, Validation. XR: Data curation, Writing – review & editing, Methodology. YW: Data curation, Writing – review & editing, Validation. XC: Data curation, Formal analysis, Investigation, Methodology, Writing – original draft. SW: Writing – review & editing, Conceptualization, Software, Validation. RY: Funding acquisition, Resources, Supervision, Writing – original draft, Writing – review & editing. JL: Funding acquisition, Resources, Supervision, Writing – original draft, Writing – review & editing, Validation. YtL: Funding acquisition, Resources, Supervision, Writing – original draft, Writing – review & editing. YeL: Funding acquisition, Resources, Supervision, Validation, Visualization, Writing – original draft. JC: Funding acquisition, Resources, Supervision, Writing – original draft.
