# Supplementary material for: Gut microbiota, circulating inflammatory proteins and sepsis: a bi-directional Mendelian randomization study
Source: Front Cell Infect Microbiol. 2024 Aug 8;14:1398756. doi: 10.3389/fcimb.2024.1398756 (PMC11338885; doi:10.3389/fcimb.2024.1398756)
Supplement: Supplementary file 2 [file DataSheet_2.docx]

**Figure S1.** MR leave-one-out sensitivity analysis for Gut microbiota on sepsis.





**Figure S2.** MR leave-one-out sensitivity analysis for Gut microbiota on sepsis (28-day mortality in critical care units)





**Figure S3.** MR leave-one-out sensitivity analysis for Gut microbiota on sepsis (critical care units)


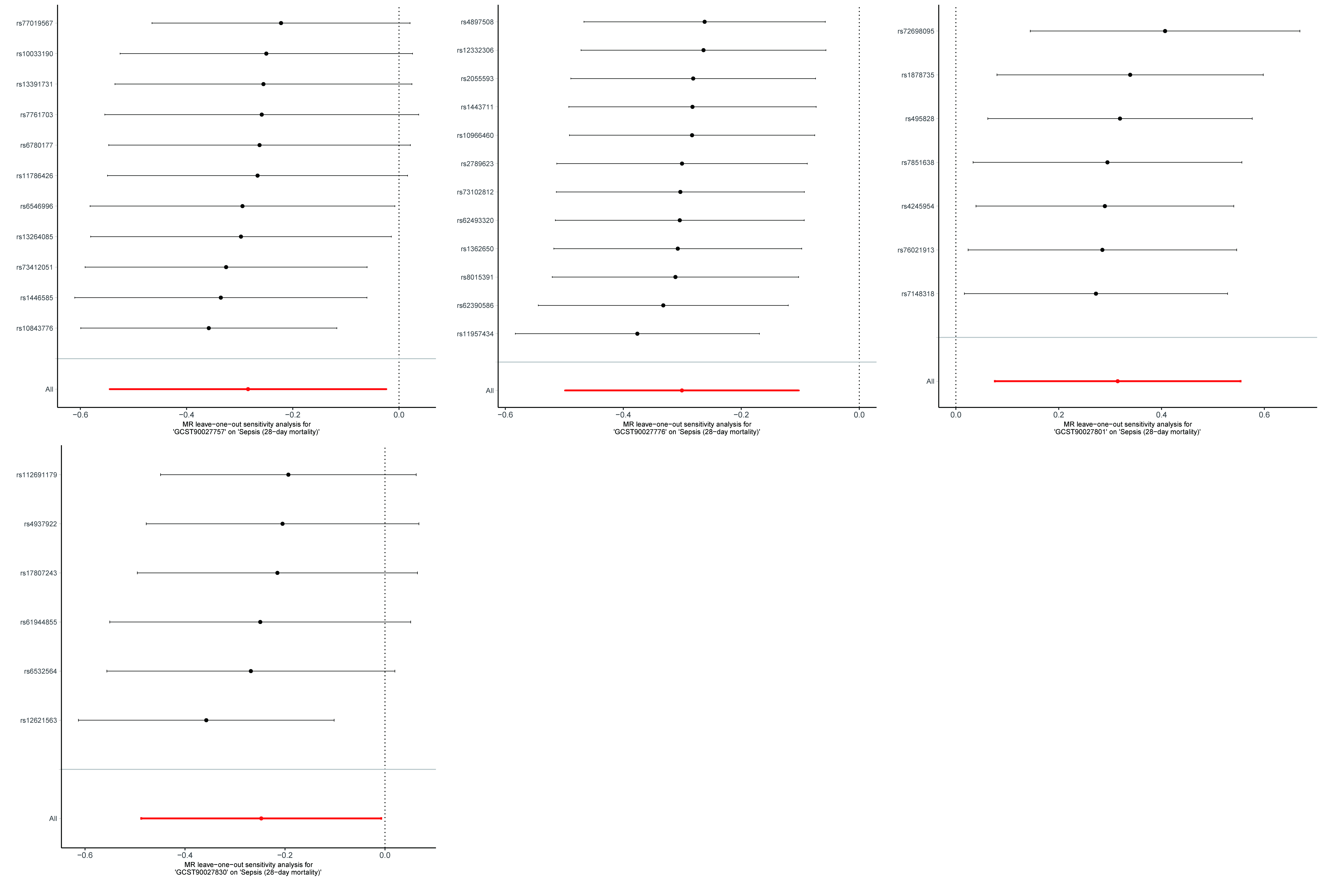


**Figure S4.** MR leave-one-out sensitivity analysis for Gut microbiota on sepsis (28-day mortality)


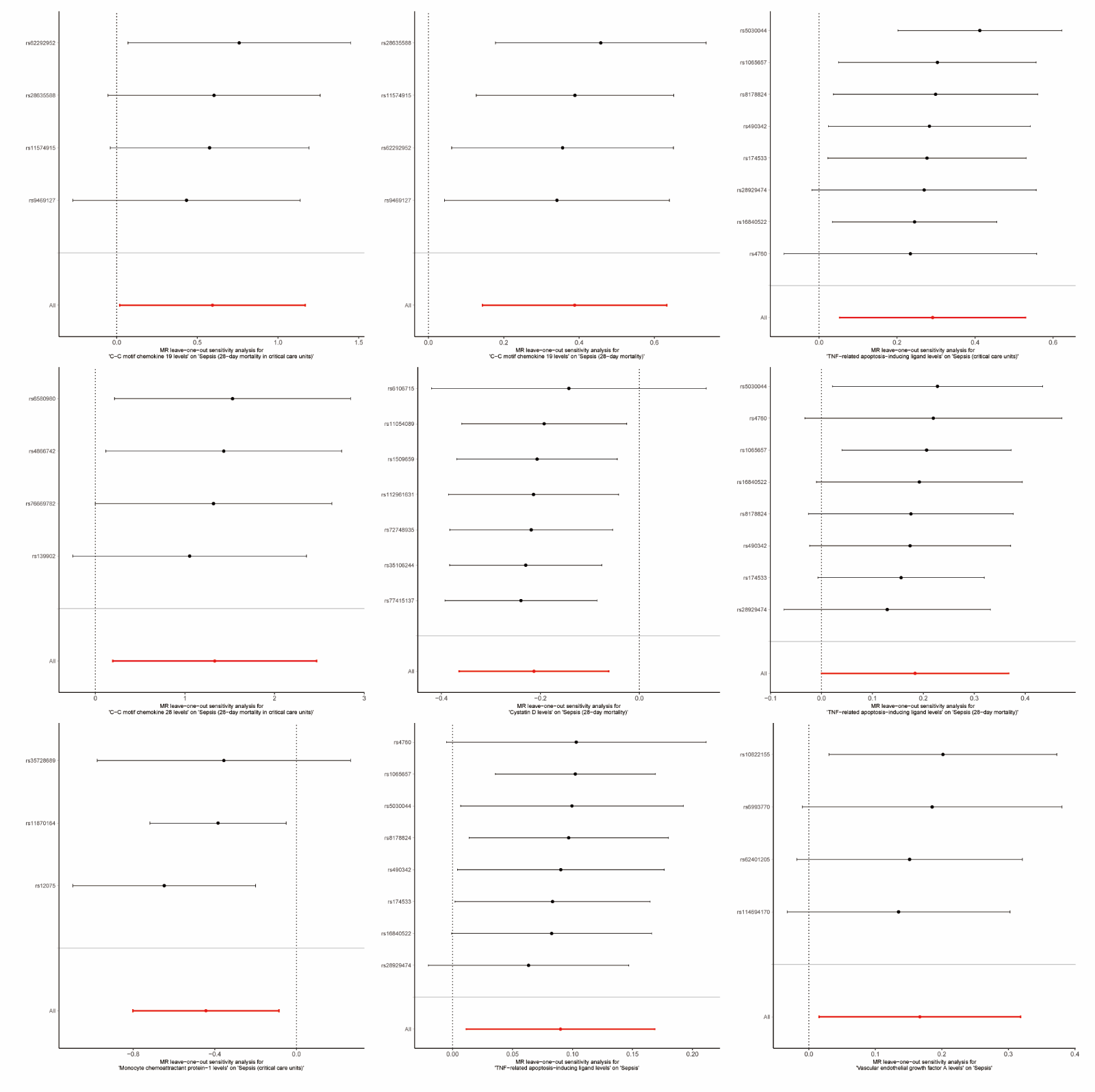


**Figure S5.** MR leave-one-out sensitivity analysis for circulating inflammatory proteins on four sepsis-related outcomes
